# 

**Published:** 1914-02

**Authors:** 


### CONTENTS.

ORIGINAL COMMUNICATIONS—

The X-Ray In Diagnosis of Diseases of the Stomach—With Lantern Slide Demonstration, by Dr. L. Amster. Diseases of the Digestive Organs and Metabolism, (Piedmont Sanatorium), Atlanta, Ga. ..... 479

Present Day Faith In Sputum Examination In Its Relation to Pulmonary Tuberculosis, by A. B. Elkin, M. D., Atlanta, Ga., Lecturer on Physical Diagnosis, Atlanta Medical College ..... 486

Child-Birth: Perineal Tears, Forceps, Chloroform, Massage. By E. C. Cartledge, M. D., Atlanta, Ga. ..... 489

A Few Brief Remarks As Regards the Psychosis of Alcohol and Morphine, by J. Cheston King, A. B., M. D. Atlanta, Ga. ..... 492

Report of Cases, by R. R. Kime, M. D., Atlanta, Ga. 1—Carbolic Acid Poisoning—1 Tablespoonful—Recovery. II—Tonsilitis followed by Nephritis and Convulsions. III—Fulminating Appendicitis—Abscess, Peritonitis—Cesorean Section ..... 495

Report of Case of Cancer of the Tongue Associated With Syphilis, by James L. Campbell, M.D., Atlanta, Ga. ..... 501

Regular Meeting Fulton County Medical Society, February 5, 1914. Reported by R. R. Daly, M. E., ..... 503

Surgery at the Panama-Pacific International Exposition ..... 507

EDITORIALS ..... 517

